# A novel function of AAA-ATPase p97/VCP in the regulation of cell motility

**DOI:** 10.18632/oncotarget.27419

**Published:** 2020-01-07

**Authors:** Zi-Jia Khong, Soak-Kuan Lai, Cheng-Gee Koh, Susana Geifman-Shochat, Hoi-Yeung Li

**Affiliations:** ^1^School of Biological Sciences, College of Science, Nanyang Technological University, Singapore 637551, Singapore

**Keywords:** p97/VCP, AAA-ATPase, ROCK, RhoA, cell motility

## Abstract

High level of the multifunctional AAA-ATPase p97/VCP is often correlated to the development of cancer; however, the underlying mechanism is not understood completely. Here, we report a novel function of p97/VCP in actin regulation and cell motility. We found that loss of p97/VCP promotes stabilization of F-actin, which cannot be reversed by actin-destabilizing agent, Cytochalasin D. Live-cell imaging demonstrated reduced actin dynamics in p97/VCP-knockdown cells, leading to compromised cell motility. We further examined the underlying mechanism and found elevated RhoA protein levels along with increased phosphorylation of its downstream effectors, ROCK, LIMK, and MLC upon the knockdown of p97/VCP. Since p97/VCP is indispensable in the ubiquitination-dependent protein degradation pathway, we investigated if the loss of p97/VCP hinders the protein degradation of RhoA. Knockdown of p97/VCP resulted in a higher amount of ubiquitinated RhoA, suggesting p97/VCP involvement in the proteasome-dependent protein degradation pathway. Finally, we found that p97/VCP interacts with FBXL19, a molecular chaperone known to guide ubiquitinated RhoA for proteasomal degradation. Reduction of p97/VCP may result in the accumulation of RhoA which, in turn, enhances cytoplasmic F-actin formation. In summary, our study uncovered a novel function of p97/VCP in actin regulation and cell motility via the Rho-ROCK dependent pathway which provides fundamental insights into how p97/VCP is involved in cancer development.

## INTRODUCTION

The Valosin Containing Protein (VCP), also known as p97, is one of the most extensively studied type II ATPase Associated with a variety of Activities (AAA) [1, 2]. As its name suggests, the AAA protein is involved in a wide range of cellular events including ubiquitin-associated protein degradation, endoplasmic reticulum-associated protein degradation (ERAD) pathway, membrane homeostasis, cell cycle regulation, the maintenance of DNA integrity, apoptosis and transcription [3–10]. Most of p97/VCP’s uncovered functions are attributed to its close associations with cellular protein degradation pathways [11–14]. In recent reports, altered p97/VCP expression was reportedly observed in specific cancer cells. For example, the expression level of p97/VCP is drastically elevated in a myriad of carcinomas and is usually correlated with poor survival probabilities [4].

The actin cytoskeleton plays a major role in defining the potential of a cell’s motility [15]. The dynamic polymerization and depolymerization of actin molecules, contractility of the actin network and the dynamic reorganization of actin scaffold are some key events that maintain the proper migration status in cells [16]. Rho-associated protein kinase (ROCK), is a serine/threonine kinase that is known to promote actin polymerization and the formation of stable filamentous actin [17]. One of the most well-established pathways that involve ROCK signaling is the Rho-ROCK signaling pathway. The Rho-ROCK pathway is central to actin cytoskeleton homeostasis through the phosphorylation of the ROCK protein’s downstream targets [18, 19]. Increased ROCK activity generally favors stress fibers formation and stabilization of filamentous actin structures [20].

ROCK signaling is implicated in the control of cell motility, and it is integral in physiological processes such as wound healing and cell migration [21]. A hallmark of malignant cancers is the metastasis of cancer cells from primary sites to distal organs [22]. Most cancer deaths are a result of the development of metastatic properties in tumors [23]. Understanding the mechanism that drives the uncontrolled migration of cancer cells is necessary for the prospect of an effective treatment against deadly cancers [24]. The process of metastasis is driven, in large part, by abnormal alterations in the cell migration properties of cells. Consistently, there are several groups proposing ROCK as a potential target in cancer therapy [25, 26]. Likewise, in wound healing, regulating ROCK activity may favor the regeneration of human organs that proves useful in degenerative or disease therapy.

In this paper, we found that p97/VCP is necessary for the maintenance of the actin cytoskeleton morphology and addressed the molecular mechanisms affected by the loss of p97/VCP. We showed that p97/VCP is implicated in cell migration, through alterations in the Rho-ROCK pathway. Together, our results revealed a novel functional role of p97/VCP in the regulation of cell migration and its underlying molecular mechanism. More importantly, we found that p97/VCP may be implicated in cancer cell migration properties, which are consistent with prominent findings stating that an increase in p97/VCP expression positively correlates with the invasiveness of most tumor cells [4].

## RESULTS

### p97/VCP regulates F-actin formation

**Figure 1:**
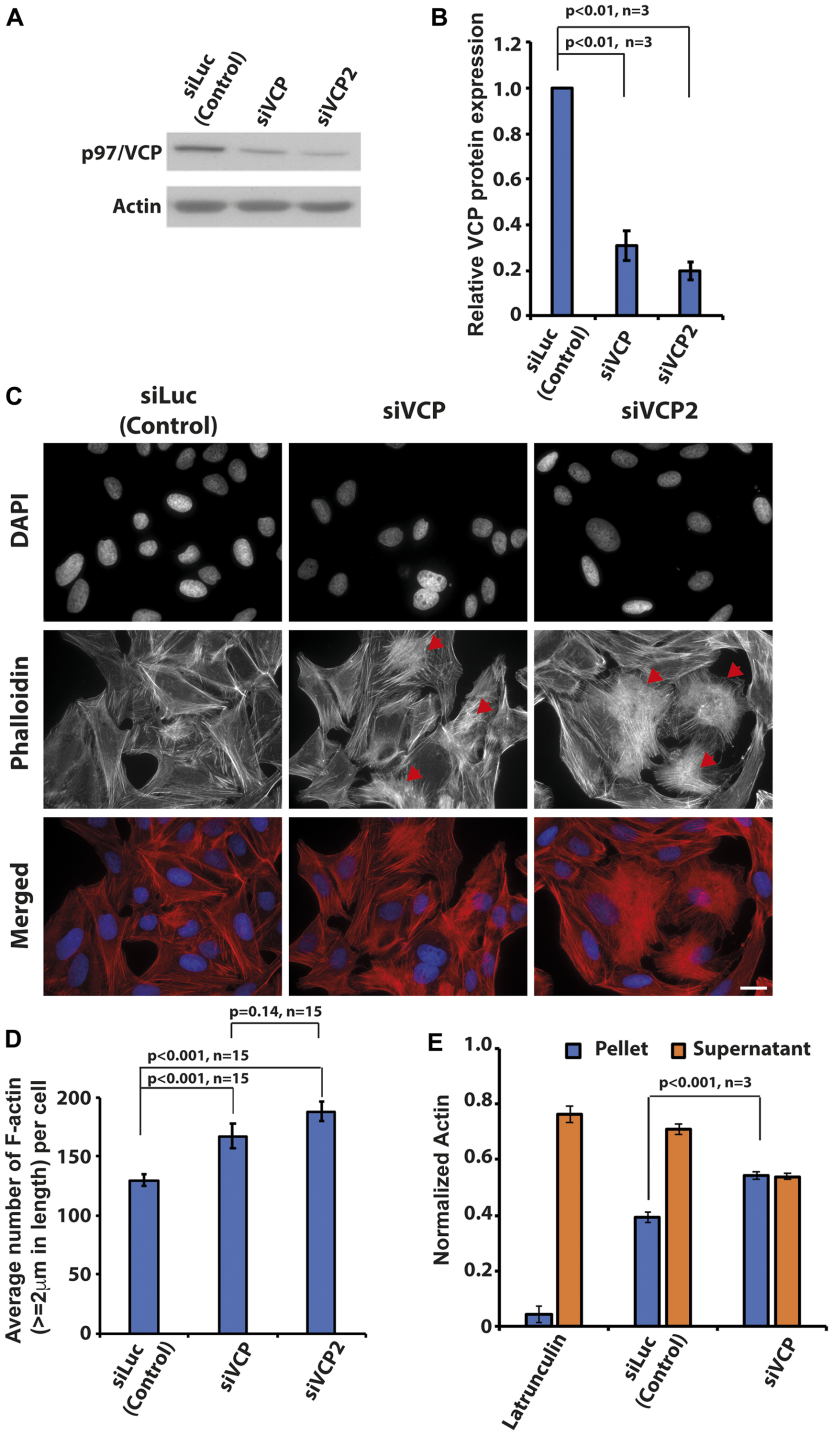
Knockdown of p97/VCP induced changes in actin morphology. (**A**) Two siRNA against p97/VCP (siVCP and siVCP2) were used in the study. At 48 hours post-transfection, both siVCP and siVCP2 were analyzed by western blot. (**B**) Both siRNAs were able to reduce p97/VCP protein levels significantly (*n* = 3 from 3 independent experiments, *p*-values are indicated in the histogram and error bars show ± SEM). (**C**) U-2 OS cells depleted of p97/VCP and control cells were stained by Phalloidin and DAPI to visualize F-actin and nucleus, respectively. p97/VCP knockdown cells showed increased amount of F-actin formation as compared with control. Red arrowheads indicate heavily polymerized F-actin. Scale bar = 10 µm. (**D**) The histogram shows the Image J quantification of the number of F-actin (actin length ≥ 2 um) in both p97/VCP knockdown and control cells (*n* = 15 from 3 independent experiments, *p*-values are indicated in the histogram and error bars show ± SEM). (**E**) Quantitative differential ultracentrifugation showed that there was an increased F-actin and decreased G-actin in p97/VCP knockdown cells. (*n* = 3 from 3 independent experiments, *p*-value is indicated in the histogram and error bars show ± SEM).

Increased p97/VCP expression accounts for enhanced survivability in many cancers including osteosarcoma [4, 27]. To understand the physiological roles of p97/VCP in cancer, we performed a siRNA knockdown of p97/VCP and screened for morphological abnormalities in U-2 OS. We used two different siRNA oligos to knockdown p97/VCP, both achieving the overall knockdown efficiency of about 70% as compared with endogenous p97/VCP protein (Figure 1A and 1B). Surprisingly, we found that the loss of p97/VCP resulting from independent siRNA knockdowns led to increased F-actin formation, which was not observed in siLuciferase-transfected cells (Figure 1C). To confirm this, we quantified the number of F-actin in both p97/VCP and control knockdown cells using Image J (Ridge-detection plugin) with the same threshold for all samples. Consistently, there was a significantly higher number of F-actin (actin length ≥ 2 um were counted) in p97/VCP knockdown cells compared with control cells (Figure 1D). Furthermore, we performed quantitative differential ultracentrifugation, probing for the levels of F and G actin in control, and p97/VCP knockdown cells. The results were also consistent with phalloidin-stained cells showing increased F-actin and decreased G-actin in p97/VCP knockdown cells (Figure 1E). These findings provided a hint that p97/VCP may be involved in the regulation of proper cellular actin morphology, an undefined function of p97/VCP.

### Cells lacking p97/VCP were more resistant to the disruptive effects of Cytochalasin D

**Figure 2:**
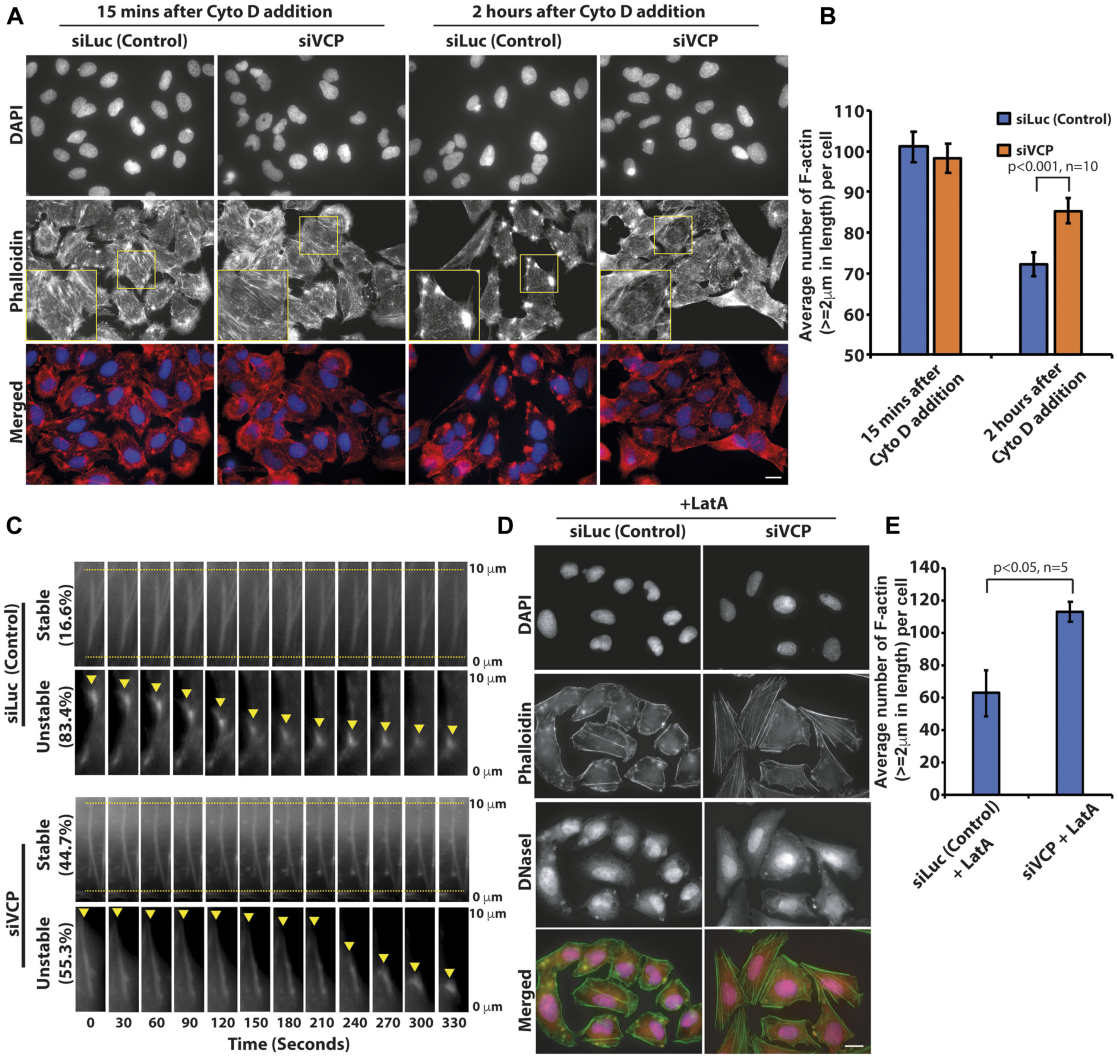
p97/VCP knockdown reduced the actin-depolymerizing effect of Cytochalasin D. (**A**) Both control and p97VCP knockdown U-2 OS cells were treated with cytotoxin Cytochalasin D and subsequently stained with Phalloidin. siVCP-treated cells were more resistant to the actin-destabilizing effects of Cytochalasin D compared to control cells. Cells retained some F-actin polymers despite prolonged treatment with Cytochalasin D. Scale bar = 10 µm. (**B**) The histogram shows the Image J quantification of the number of F-actin (actin length ≥ 2 um) in both p97/VCP knockdown and control cells after Cytochalasin D treatment. (*n* = 10 from 3 independent experiments, *p*-value is indicated in the histogram and error bars show ± SEM. (**C**) Kymograph showing shrinkage of actin filaments at the minus end (yellow arrowhead) upon treatment with Cytochalasin D in siVCP-treated U-2 OS cells. Actin-GFP was imaged immediately after cells were treated with cytotoxin Cytohalasin D for 5 minutes. In control cells, over 80% of actin filaments depolymerized in a short time, only about 16% of actin filaments remained stable without depolymerization during the live cell imaging experiment. In contrast, 44.7% of actin remained stable in p97/VCP knockdown cells. Cells were treated with Cytochalasin D and imaged five minutes post drug treatment. The percentages of the proportion of actin filaments, which were either stable or unstable, are denoted by the values on the left of the kymograph. Ten actin filaments were analyzed within each of the five cells in each experiment. Representative images were obtained across three independent time-lapse experiments. (**D**) Both control and p97VCP knockdown U-2 OS cells were treated with Latrunculin A and subsequently probed with fluorescence-conjugated Phalloidin and DNaseI. siVCP-treated cells were more resistant to the actin-destabilizing effects of Latrunculin A compared to control cells. Scale bar = 10 µm. (**E**) The histogram shows the Image J quantification of the number of F-actin (actin length ≥ 2 um) in both p97/VCP knockdown and control cells after Cytochalasin D treatment. (*n* = 5 from 3 independent experiments, *p*-value is indicated in the histogram and error bars show ± SEM).

Since the loss of p97/VCP enhanced F-actin formation, we tested if p97/VCP knockdown cells were able to counter the depolymerizing effects of Cytochalasin D on the F-actin. Cytochalasin D is a cytotoxin commonly used to inhibit actin polymerization and its detrimental effects on actin dynamics block proper cell migration [28]. We treated control and p97/VCP-knockdown cells with Cytochalasin D and visualized the resulting actin morphology. The fast-acting inhibitory mechanism of Cytochalasin D caused the formation of actin aggregates and foci within 15 minutes of drug treatment in control cells. In contrast, cells lacking p97/VCP were more resistant to the damaging effects of Cytochalasin D and the formation of actin aggregates and foci were less evident even after prolonged drug treatment (Figure 2A, 2B and Supplementary Figure 1A). It appears that the loss of p97/VCP resulted in the enrichment of highly stable, polymeric F-actin and therefore was able to counteract the inhibitory effect of Cytochalasin D on actin polymerization.

To further characterize the changes in actin behavior upon treatment with Cytochalasin D, we performed time-lapse microscopy monitoring real-time depolymerization of actin filament bundles during drug action in actin-GFP expressing cells. In accordance to results obtained from immunostaining, cells lacking p97/VCP had enrichment of stable F-actin that did not depolymerize through the course of the entire time-lapse. In each observed siLuc-treated cells, over 80% of the F-actin exhibited steady shrinkage, eventually collapsing into the oligomeric actin nucleus. On the other hand, over half of the siVCP-treated cells appeared stable at the initial phase but rapidly depolymerized as the drug exposure period increases. The other half of the population of actin filaments was stable and did not depolymerize throughout the entire time-course (Figure 2C and Supplementary Figure 1B). This data shows that the stabilizing effect seen upon the loss of p97/VCP is unlikely to be due to increased actin polymerization at the barbed ends of the actin filaments.

We also repeated the experiment using Latrunculin A. The addition of Latrunculin A reduced the number and intensity of actin filaments in control cells. In contrast, there was almost twice the number of stable F-actin filaments present in p97/VCP knockdown cells treated with Latrunculin A. We used fluorescence-conjugated Deoxyribonuclease I to concurrently visualize unpolymerized G-actin upon treatment with Latrunculin A (Figure 2D and 2E). For F-actin to remain stable during treatment with Cytochalasin D, capping proteins must compete with Cytochalasin D for binding to the (+) end. On the other hand, for F-actin to remain stable during treatment with Latrunculin A, there must be reduced spontaneous dissociation from the (–) end. Thus, p97/VCP is involved in multiple pathways to regulate actin dynamics.

### Enhanced F-actin formation upon p97/VCP silencing hinders proper cell migration

**Figure 3:**
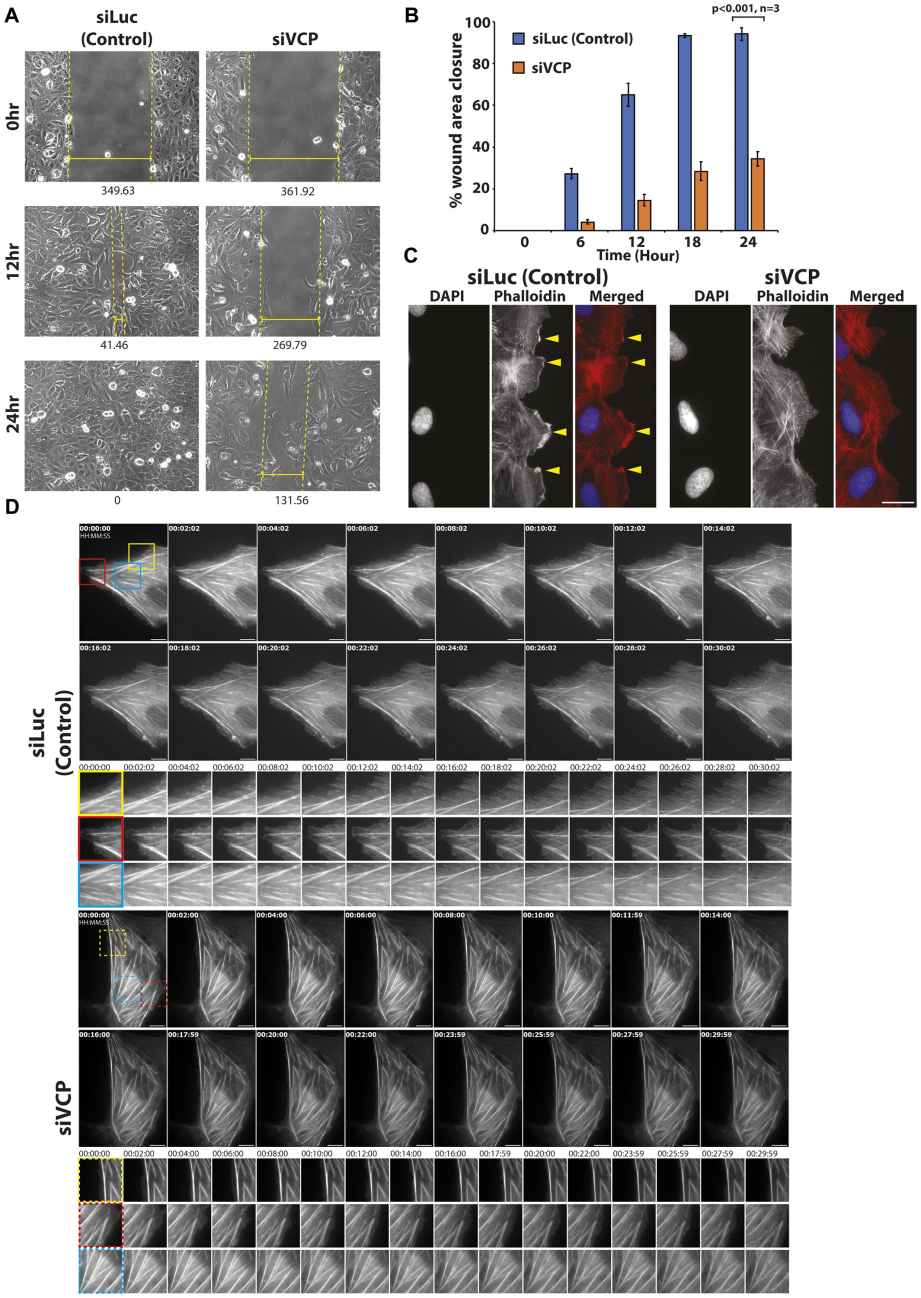
Knockdown of p97/VCP impaired proper cell migration. (**A**) Migration abilities of U-2 OS cells were observed in a scratch wound assay over a time course of 24 hours. Glass bottom dishes were scratched, and time-lapse images were taken over a time course. After 12 hours upon wounding, siLuc-treated cells have migrated efficiently towards the site of wounding. At 24 hours, the wound has fully healed. In siVCP-treated cells, migration was impaired, and minimal wound healing was observed. Numbers below images represent the width (nm) of the wound, and each image was obtained using Axiovision. Images were taken using 20× Phase contrast objective. (**B**) Scratch wound assay was analyzed by Image J (MRI wound healing tool plugin). Histogram shows the percentage of wound closure in the time-lapse experiments (*n* = 3 from 3 independent experiments, error bars show ± SEM). (**C**) In control U-2 OS cells, there was distinctive formation of lamellipodia at the leading edge of migrating cells (yellow arrowheads). Thin actin filaments were also observed. In siVCP knockdown cells, there was no clear formation of lamellipodia in migrating cells. (**D**) Live cell-imaging of control and siVCP knockdown U-2 OS cells showing the difference in actin dynamics in the presence and absence of p97/VCP. In control cells, actin filament bundles are dynamic while in siVCP knockdown cells, most filament bundles were static over the course of the time-lapse. Scale bar = 10 µm. Color boxes are enlarged images of the movie.

It is established that coordinated actin morphology and dynamics are crucial to proper cell migration [29]. Since we observed more filamentous F-actin in p97/VCP knockdown cells, we were interested in examining its effects on cell migration properties. To do so, we performed a scratch wound assay followed by time-lapse microscopy to observe cell movement towards the site of wounding. Interestingly, results of the scratch wound assay show that loss of p97/VCP led to impairments in cell migration compared to cells treated with control siRNAs, (Figure 3A, Supplementary Figure 2A). Quantitative analyses using Image J (MRI wound healing tool plugin) showed that the area of wound closure of p97/VCP-knockdown cells was significantly lower than in control cells (Figure 3B). Similarly, in HeLa cells, p97/VCP knockdown cells were less adept in wound closure compared to control cells (Supplementary Figure 2B). These results suggest that p97/VCP knockdown cells migrated at a significantly slower rate and were unable to close the inflicted scratch wound fully.

To determine the cause of the defective migration abilities observed in p97/VCP knockdown cells, we examined the actin morphology of actively migrating cells. First, a wound is inflicted like before and allowed for wound healing. Cells were then fixed prior to complete wound healing and stained for Phalloidin to visualize F-actin filaments in cells at the leading edge of the wound. The formation of these dynamic actin assemblies at the leading edge of actively migrating cells are necessary for proper cell migration. We observed distinct lamellipodia-like structures at the leading edges of normal migrating cells (Figure 3C, yellow arrowheads). On the other hand, in cells treated with p97/VCP siRNAs, there was no obvious formation of the polarized leading edge or the lamellipodia (Figure 3C). The lack of these essential cytoskeletal actin components may contribute to the defective migration abilities of p97/VCP-deficient cells.

To determine the cause of the compromised migration abilities observed in p97/VCP knockdown cells, we studied the actin dynamic of actively migrating cells using live-cell imaging. We showed in real-time, the difference in actin dynamics in control and p97/VCP-deficient cells. In control cells, there is dynamic actin activity at the cell periphery (filopodia, lamellipodia, and actin fiber formation). However, in p97/VCP knockdown cells, most actin filament bundles were stable and static over the course of the time-lapse imaging (Figure 3D, Supplementary Figure 3, Supplementary Movie 1). The lack of these essential cytoskeletal actin components may contribute to the defective migration of p97/VCP-deficient cells. p97/VCP knockdown cells may be lacking in actin-related structures necessary for proper cell migration, further highlighting the involvement of p97/VCP in cytoskeletal maintenance.

### Extensively polymerized actin in p97/VCP knockdown cells is due to Rho-ROCK dependent pathway

**Figure 4:**
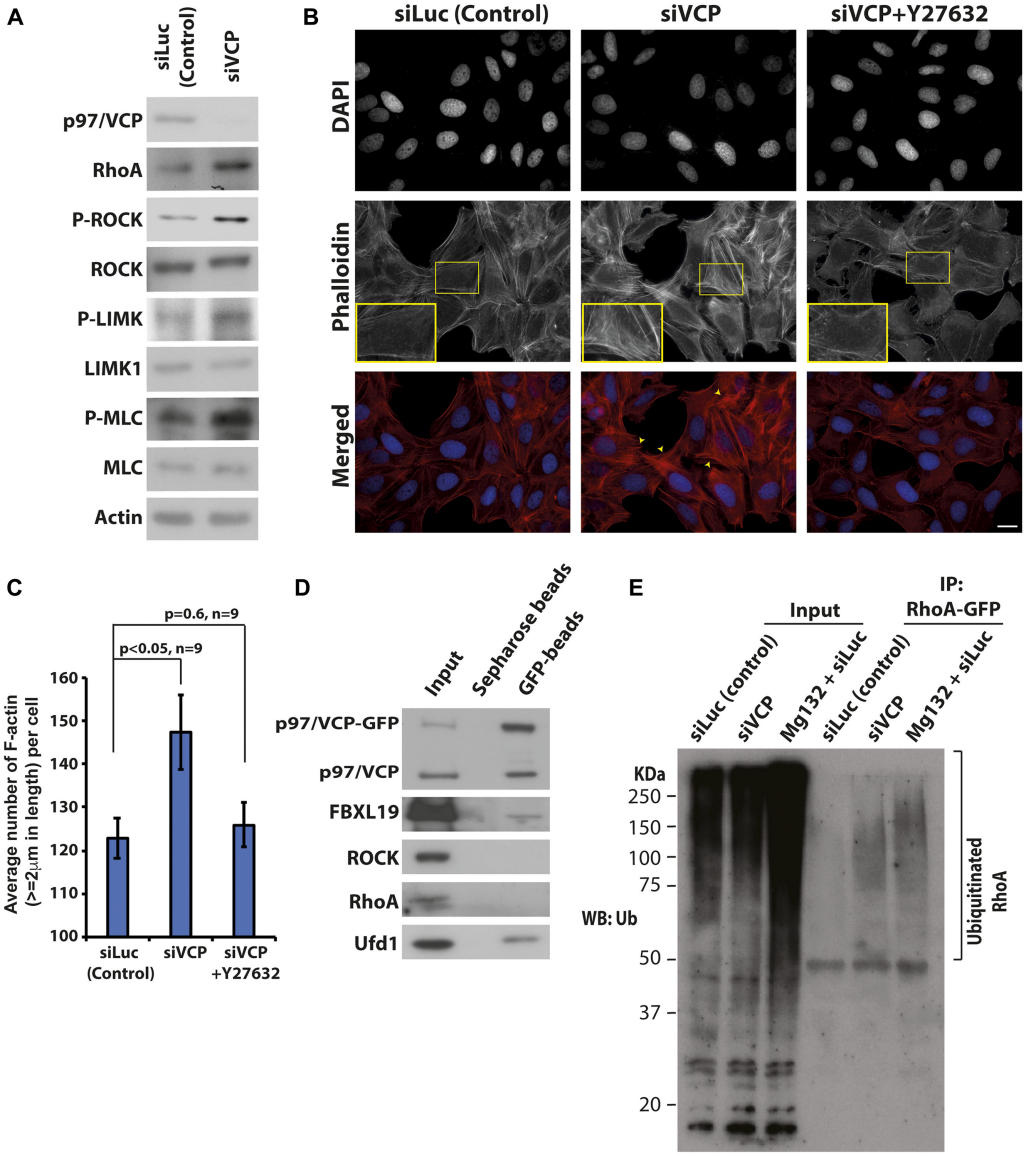
Loss of p97/VCP induces Rho-ROCK signaling pathway. (**A**) Whole-cell lysates were prepared from U-2 OS cells transiently transfected with control siLuc and p97/VCP siRNA. Western bolt was carried out to analyze the major proteins in the Rho-ROCK signaling pathway. (**B**) Phalloidin staining of U-2 OS cells was performed to visualize F-actin upon the knockdown of p97/VCP and after treatment with Y-27632. Short treatment with Y-27632 effectively rescued the aberrant phenotype observed in siVCP knockdown cells. Scale bar = 10 µm. (**C**) The histogram shows the Image J quantification of the number of F-actin (actin length ≥ 2 um) in p97/VCP knockdown, p97/VCP knockdown with Y-27632 treatment and control siLuc treated cells. (*n* = 9 from 3 independent experiments, *p*-values are indicated in the histogram and error bars show ± SEM. (**D**) Co-immunoprecipitation of p97/VCP-GFP showed interaction with FBXL19. p97/VCP cofactor, Ubiquitin fusion protein degradation 1 (Ufd1) was used as a positive control. (**E**) RhoA-GFP was ubiquitinated in the absence of p97/VCP. U-2 OS cells were treated with either siLuc (siLuc and Mg132) or siVCP siRNA 72 hours prior to cell lysis. RhoA-GFP was transfected 24 hours prior to cell lysis. RhoA-GFP was immunoprecipitated using anti-GFP antibody conjugated agarose beads and analyzed by the anti-Ubiquitin antibody. Mg132 treated samples were used as a positive control.

One of the best-characterized regulators of actin dynamics is the Rho GTPase signaling pathway. The proteins involved in the Rho-dependent signaling cascade has been well established, many of which are regulated by phosphorylation [30, 31]. Since proteins of the Rho pathway are responsible for actin dynamics required for cell migration, we looked for possible changes in the expression levels and phosphorylation statuses of these proteins upon p97/VCP knockdown. Upon knockdown of p97/VCP, there was an increase in RhoA level coupled with increased phosphorylation of its downstream effectors, ROCK, LIMK, and MLC proteins (Figure 4A, Supplementary Figure 4). This suggests that the enhanced F-actin architectures and diminished cell migration capabilities in p97/VCP knockdown cells are regulated by Rho-ROCK dependent pathway.

To further confirm this, we treated p97/VCP-knockdown cells with a potent and selective inhibitor of ROCK, Y-27632. Y-27632 inhibits the kinase activities of both ROCKI and ROCKII in a competitive manner, resulting in diminished F-actin formation [32]. As expected, most of the F-actin was diminished in p97/VCP-knockdown cells treated with Y-27632 (Figure 4B and 4C). Hence, the enrichment of actin filaments upon the loss of p97/VCP can be attributed to enhanced ROCK activity since treatment with Y-27632 rescued the observed phenotype.

### p97/VCP regulates ubiquitination dependent RhoA degradation

To understand how p97/VCP regulates the Rho pathway proteins, we performed co-immunoprecipitation to investigate if p97/VCP interacts with key players of the Rho pathway. It appears that p97/VCP does not interact directly with Rho or ROCK proteins. Interestingly, we found that p97/VCP interacts with FBXL19 (Figure 4D). FBXL19 is an F-box protein within the Skp1-Cul1-F-box protein (SCF) ligase complex that regulates RhoA stability by specifically targeting ubiquitinated RhoA for degradation. A reduction in FBXL19 activity was found to increase the half-life of RhoA and RhoA cellular protein levels [33]. As the targets of p97/VCP are commonly chaperoned and degraded by the Ubiquitin-Proteasome System (UPS) [34], it is possible that p97/VCP acts as a molecular chaperone to guide FBXL19 targets for proteasomal degradation. Therefore, the loss of p97/VCP may lead to the increased protein level of RhoA, which in turns phosphorylate and activate ROCK proteins and other downstream effectors. Hence, we examined whether knockdown of p97/VCP would result in the accumulation of ubiquitinated RhoA. RhoA-GFP was expressed in p97/VCP knockdown cells, control siRNA cells and cells treated with MG132 (as positive control). RhoA-GFP was then pulled down by anti-GFP bead followed by western blot analysis using anti-ubiquitin antibody. As shown in Figure 4E, ubiquitinated RhoA was enriched in p97/VCP siRNA knockdown cells and MG132 treated cells but not in control knockdown cells.

## DISCUSSION

In summary, our proposed model entails the overall cell homeostasis system surrounding p97/VCP, RhoA, ROCK proteins, and F-actin. Under physiological conditions, p97/VCP oversees the strict balance in the protein homeostasis of RhoA. Reduction of p97/VCP results in an enrichment of RhoA protein, which in turn enhanced the formation of F-actin and subsequent decrease of the cell motility by activating ROCK, LIMK, and MLC proteins. This pathway is quintessential in the fundamental process of cell migration in normal cells. One hallmark of cancer is, in fact, the uncontrolled cell migration from the tumor site. Many groups have since found that p97/VCP is indisputably upregulated in most cancers. Consistently, our model suggests that in cancer cells where p97/VCP is more abundant, RhoA level turnover is misregulated. As such, ROCK proteins are less active leading to dynamic actin network formation and ultimately, increased cell migration potential. A recent study also reported that a chemical inhibitor of p97/VCP also inhibited mouse fibroblast cell migration [35].

The perturbations in actin dynamics in p97/VCP-knockdown cells suggest that p97/VCP might have a role in the regulation of cell migration. Actin protrusive structures are critical in driving proper cell movement, particularly in cancerous cells [36–38]. Consistent with our hypothesis, results from our scratch wound assays demonstrated that cells lacking p97/VCP were less migratory coupled with the loss of motility-related actin protrusive structures, such as lamellipodia. It is noteworthy that Osteosarcoma cancer cells display high metastatic characteristics and is especially relevant in the study of cancer metastasis [27]. The apparent loss of migratory abilities suggests that p97/VCP might have a role in the regulation of cell migration.

While it is widely known that Cytochalasin D caps the barbed ends of actin filaments, preventing both the polymerization and depolymerization of actin monomers at one end [39, 40]. Nonetheless, spontaneous dissociation of actin monomers still may occur at the pointed ends of actin polymers, leading to a net shortening of actin filaments upon treatment with Cytochalasin D [39]. Accordingly, our data showed steady shortening of actin filaments at one end in control cells momentarily upon the addition of Cytochalasin D. Surprisingly, cells lacking p97/VCP with stable actin filaments did not exhibit this phenomenon in most actin filament bundles that were imaged. One possible explanation for this observation could be that the behavior of the pointed ends-capping proteins, Tropomodulins, are altered upon the loss of p97/VCP [41]. While there are four known Tropomodulin genes, TMOD1-4, TMOD1 seemed to be the ubiquitous gene expressed in the various cell lines used in our study [42]. We are still in the midst of investigating the effects of p97/VCP knockdown on TMOD1 gene and protein expressions. Another surprising observation seen in half of the imaged actin filaments was the sudden shortening of some filaments as Cytochalasin D treatment duration lengthened. While the complex mechanism of action of Cytochalasin D is still unclear, some papers report the Cytochalasin D may cause ‘severing’ of actin filaments under certain conditions [40]. It is logical since the knockdown of p97/VCP seemed to stabilize the pointed ends of actin filaments, but it may not protect against the ‘severing’ within the length of the actin polymer.

We also found that p97/VCP may participate in the induction of actin-based cytoplasmic protrusions, such as the filopodia and lamellipodia, during cell migration. It is possible that the presence of unusually stable microfilaments impeded the proper formation of these highly dynamic migration-related cytoplasmic protrusions. However, the molecular mechanism behind the loss of migratory actin-based cytoplasmic projections still remains to be elucidated. It would be logical to look into the possible involvement of p97/VCP with proteins upstream and downstream of other ras-related GTPases, such as Rac and Cdc42 [43].

It was recurrently reported that p97/VCP expression level is elevated in cancer cells [44]. It was also reported that p97/VCP levels are generally higher in serums obtained from cancer patients compared to healthy individuals [4, 45]. Current studies focus on proteostatic involvement of p97/VCP in cancer development. Deregulated p97/VCP protein level disturbs the expression of important proto-oncogenes and oncogenes, favoring the growth of cancer cells [4]. This study presents an alternative perspective on the connection between p97/VCP and cancer. In addition to the imbalances in the proteasomal pathway, p97/VCP may trigger aberrant migration of cancer cells by altering actin-related cell migratory characteristics. As such, additional considerations can be given when designing anticancer drugs against p97/VCP.

## MATERIALS AND METHODS

### Reagents and antibodies

GFP-tagged p97/VCP plasmid was constructed by inserting cloned p97/VCP DNA into pEGFP-N3 plasmid backbone. His-tagged p97/VCP cloned into pcDNA3.1 plasmid backbone was kindly provided by Dr Chia Wei Sheng [46]. siRNA against p97/VCP (see supplementary) were obtained from Dharmacon. siVCP 1: SMARTpool: ON-TARGETplus VCP siRNA (L-008727-00). siVCP 2: SMARTpool: siGENOME VCP siRNA (M-008727-01). Cytochalasin D, Latrunculin A, Mg132 and Y-27632 were purchased from Sigma-aldrich and used at a working concentration of 10 μM. Anti-ROCKI/II, Phospho-LIMK, RhoA, cofilin, LIMK1/2, Phospho-MLC antibodies were from Cell Signaling Technology. p97/VCP, Ufd1 and Ubiquitin antibodies were from Santa Cruz Biotechnology. FBXL19 antibody was from Abgent. Alexa-Fluor 594 conjugated Phalloidin and Alexa-fluor 488 conjugated Tubulin were obtained from Thermo Fisher Scientific.

### Cell culture and transfection

U-2 OS (ATCC) and HeLa (ATCC) were maintained in Dulbecco’s modified Eagle’s medium (DMEM) supplemented with 10% Fetal Bovine Serum (FBS) and 1% Penicillin Streptomycin in a humidified chamber with 5% CO_2_ at 37°C. Cells were seeded and incubated overnight prior to transfection. Transfection was performed according to Lipofectamine 3000 (Thermo Fisher Scientific) protocol and harvested for analysis after 24 hours. For experiments requiring transfection of siRNAs, cells are seeded the day before and transfected by Lipofectamine 3000. Transfected cells were incubated for 48 hours then analyzed. In experiments requiring sequential siRNA knockdown, cells were transfected 3 times with a 24-hour incubation period between each transfection for a total for 72 hours. All cell lines were tested contamination-free prior to all experiments performed.

### Differential ultracentrifugation for ratios of F and G actin

Cell lysates were collected in 200 ul of lysis buffer (50 µM PIPES pH 6.9, 50 mM NaCl, 5 mM MgCl_2_, 5 mM EGTA, 5% (v/v) glycerol, 0.1% Nonidet P-40, 0.1% Triton X-100, 0.1% Tween 20, 0.1% 2-mercapto-ethanol, 100 mM) supplemented with a cocktail of protease inhibitors. Samples were incubated at 37°C for 10 minutes and ultracentrifuged at 150,000 g for one hour at 37°C to separate the G-actin (supernatant) and the F-actin (pellet) fractions. The pellets were resuspended with 200 ul of 10 µM cytochalasin D in water to depolymerize the F-actin filament and left on ice for one hour, vortexing every 15 minutes. The resuspended solutions were then centrifuged at 2,000 g for five minutes at 4°C to collect the dissociated actin in the supernatant. The G-actin samples and F-actin samples were used immediately for western blot analyses.

### Co-immunoprecipitation of p97/VCP and western blotting

GFP-tagged p97/VCP expressing cells were harvested with Pierce IP-lysis buffer (25 mM Tris-HCl pH 7.4, 150 mM NaCl, 1 mM EDTA, 1% NP-40, and 5% glycerol) supplemented with protease and phosphatase inhibitors. Lysates were incubated with anti-GFP antibody conjugated agarose beads or unconjugated agarose beads (Chromotek) for four hours at 4°C. The immunoprecipitants were then washed three times and heated in sample buffer for downstream analyses. In western blot analysis, the resultant SDS-PAGE gel was transferred onto a nitrocellulose membrane and probed with the relevant antibodies. Each immunoblot experiment was repeated at least three times for consistency. Densitometric analyses were performed using ImageJ.

### Immunoprecipitation and ubiquitination western blotting

Exogenous RhoA-GFP was overexpressed in cells treated with siVCP, siLuc (negative control) and siLuc with MG132 treatment (positive control). Cells were harvested with Pierce IP-lysis buffer (25 mM Tris-HCl pH 7.4, 150 mM NaCl, 1 mM EDTA, 1% NP-40, and 5% glycerol). RhoA-GFP from different samples was then immunoprecipitated by anti-GFP antibody conjugated agarose beads (ChromoTek) followed by western blot analysis using anti-ubiquitin antibody (Santa Cruz).

### Immunostaining and microscopy

Cells were fixed in 4% Paraformaldehyde (Sigma-Aldrich) and permeabilized with 0.5% Triton-X-100 (USB Corp). Next, blocking was performed with 1% Bovine Albumin Serum (BSA) (Sigma-Aldrich) in PBS with Tween-20. Primary antibodies diluted in BSA were incubated for one hour at room temperature or 4°C overnight. The appropriate secondary antibodies were added for one hour at room temperature. Lastly, slides were mounted using Prolong Gold Antifade mounting media containing DAPI (Invitrogen) to visualize DNA. For time-lapse microscopy, cells were seeded onto glass-bottom dishes before transfection then placed in Zeiss Axiovert 200M microscope (Zeiss, Germany) with humidified, heated chamber at 37°C supplied with 5% carbon dioxide. Phase-contrast images were captured with 20× objective. Fluorescence, phase contrast and Differential Interference Contrast (DIC) images were captured using 20× or 63× objectives. All image analyses are done on Axiovision 4.8 program. (Zeiss, Germany).

### Scratch wound assay

Cells were grown to 100% confluency, and scratch wounds were artificially created with 1000 ml pipette tips on the culture dishes. Cell debris was removed by washing thoroughly with Phosphate Saline Buffer (PBS) and replaced with DMEM. Cells were immediately visualized under the microscope or incubated for specific periods of time. Scratch wound assays were repeated twice to obtain the presented data. Phase-contrast images were captured with 20× objective. All image analyses are done on Axiovision 4.8 program. (Zeiss, Germany).

All experiments were repeated at least 3 times independently.

## SUPPLEMENTARY MATERIALS





## Abbreviations

ATP: adenosine triphosphate
ATPase: adenosine triphosphatase
GFP: green fluorescent protein
ROCK: Rho-associated protein kinase
Ub-Pr: Ubiquitin-proteasome
VCP: Valosin containing protein
