# Supplementary material for: A novel function of AAA-ATPase p97/VCP in the regulation of cell motility
Source: Oncotarget. 2020 Jan 7;11(1):74–85. doi: 10.18632/oncotarget.27419 (PMC6967774; doi:10.18632/oncotarget.27419)
Supplement: Supplementary file 1 [file oncotarget-11-74-s001.pdf]

A novel function of AAA-ATPase p97/VCP in the regulation of cell motility

SUPPLEMENTARY MATERIALS

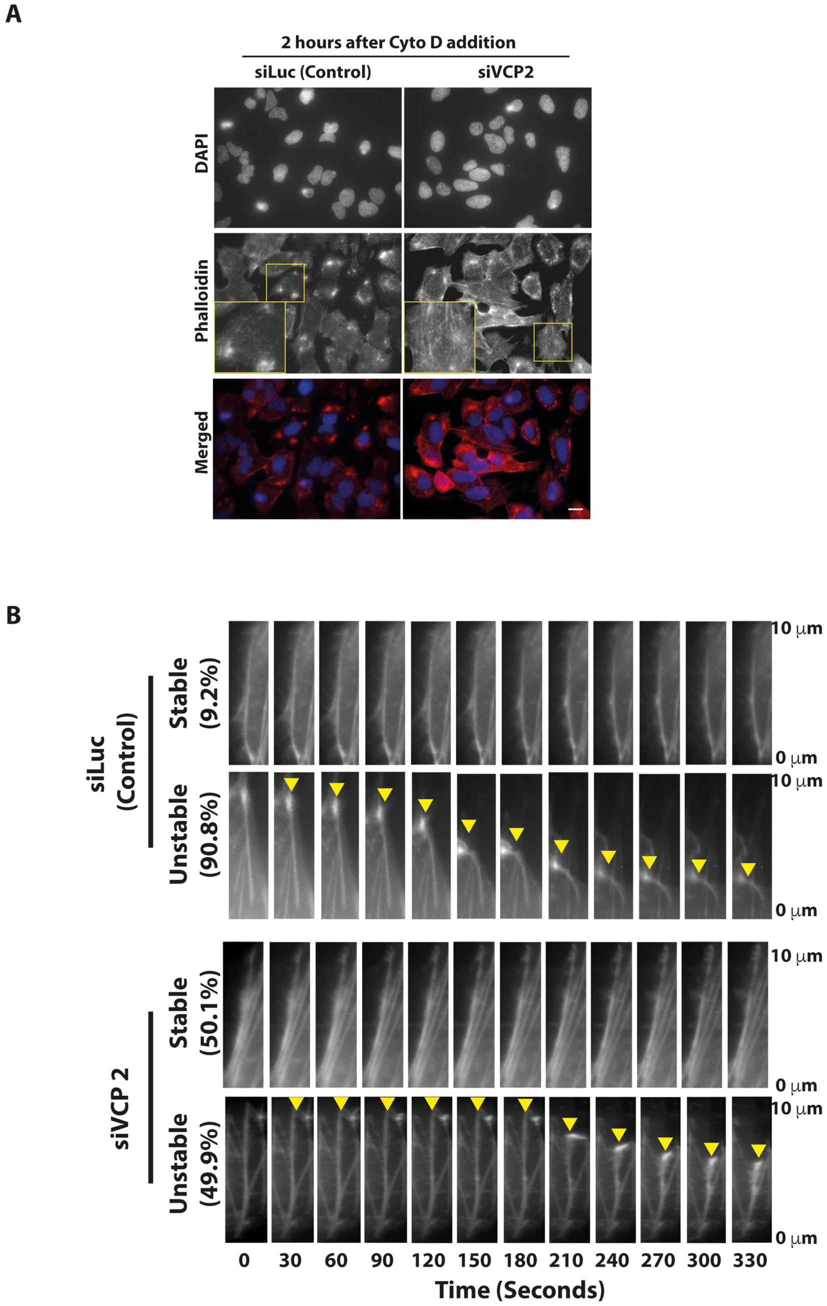

Supplementary Figure 1: Experiments performed as described in the main text and methods, repeated with a different siRNA against p97/VCP (siVCP2). (**A**). siVCP2 treated U-2 OS cells were resistant to the actin-destabilsing effects of Cytochalasin D compared with control cells. Scale bar = 10 µm. (**B**) Kymograph showing shrinkage of actin filaments at the minus end (yellow arrowhead) upon treatment with Cytochalasin D in siVCP2 treated cells. Actin-GFP was imaged immediately after cells were treated with cytotoxin Cytohalasin D for 5 minutes. In control cells, 90.8% of actin filaments depolymerized in short period of time, only about 9.2% of actin filaments remained stable without depolymerization during the live cell imaging experiment. In contrast, 50.1% of actin remained stable in p97/VCP knockdown cells. ($P < 0.001$) Cells were treated with Cytochalasin D and imaged five minutes post drug treatment.

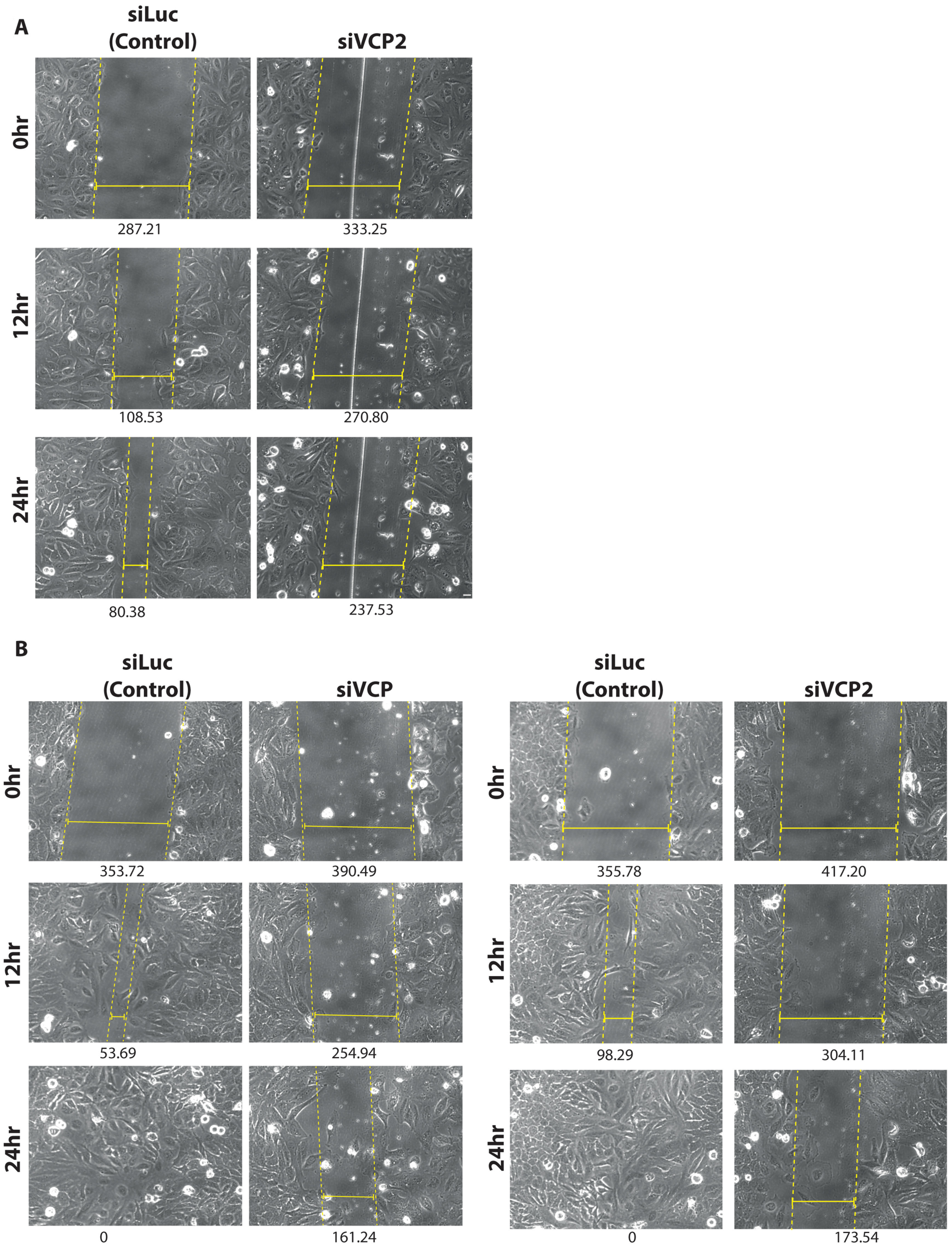

Supplementary Figure 2: (**A**) Experiment showed in Figure 3 was repeated with a different siRNA against p97/VCP on U-2 OS (siVCP2). In siVCP2-treated cells, migration was impaired and minimal wound healing was observed. Numbers below images represent the width (nm) of the wound and each image was obtained using Axiovision. Images were taken using 20x Phase contrast objective. Scale bar = 10 µm. (**B**) Experiment showed in Figure 3 was repeated with both siRNAs against p97/VCP on HeLa cells (siVCP and siVCP2). Similarly, siVCP-treated cells had impaired migration capabilities and minimal wound healing was observed. Numbers below images represent the width (nm) of the wound and each image was obtained using Axiovision. Images were taken using 20x Phase contrast objective. Scale bar = 10 µm.

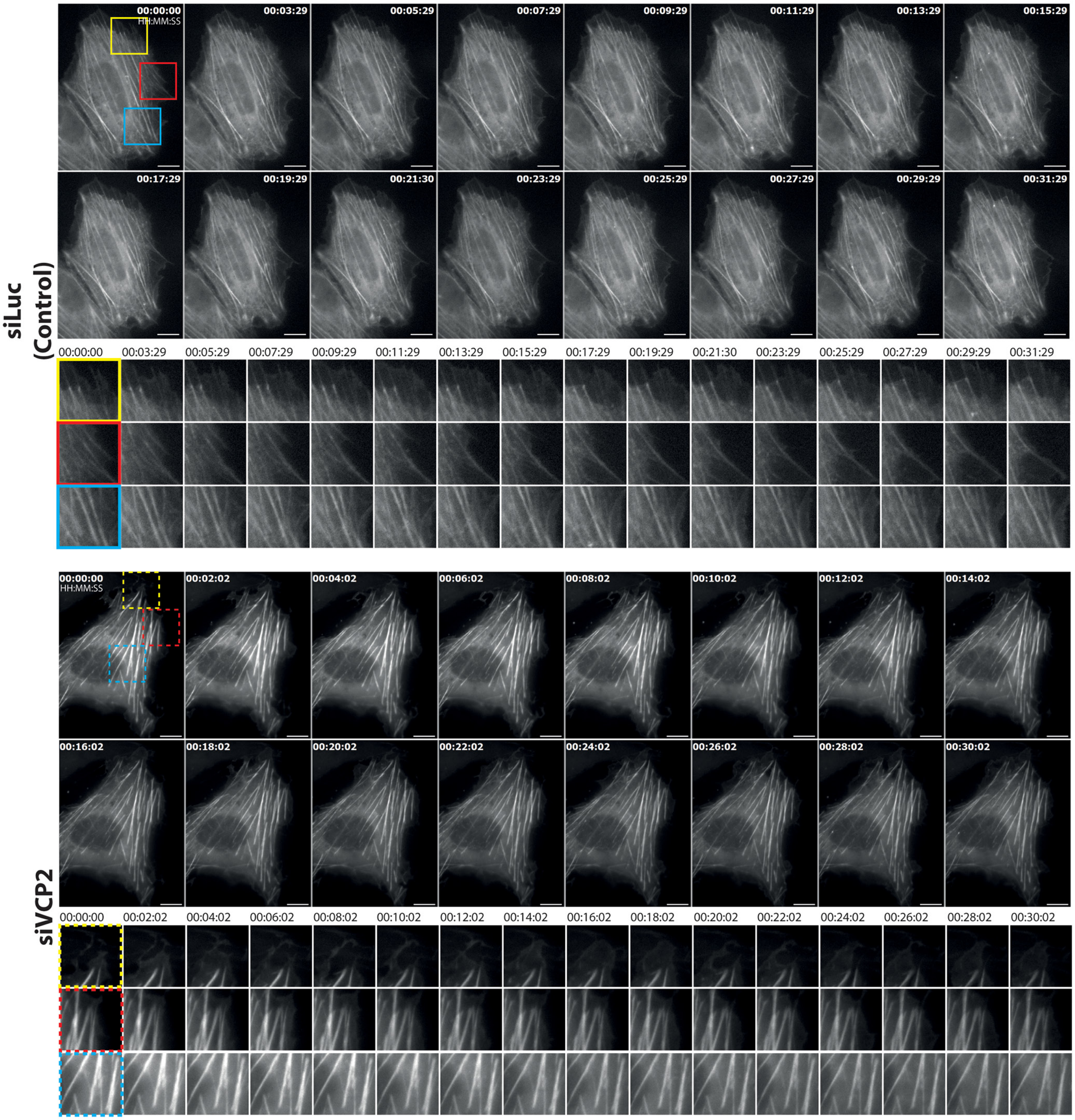

Supplementary Figure 3: Experiment showed in Figure 3C was repeated with a different siRNA against p97/VCP (siVCP2). Live cell-imaging of control and siVCP2 knockdown U-2 OS cells showing difference in actin dynamics in the presence and absence of p97/VCP. In control cells, actin filaments are dynamic while in siVCP knockdown cells, most filaments were static over the course of the time-lapse. Three independent experiments were performed. Scale bar = 10 µm. Color boxes are enlarged images of the movie.

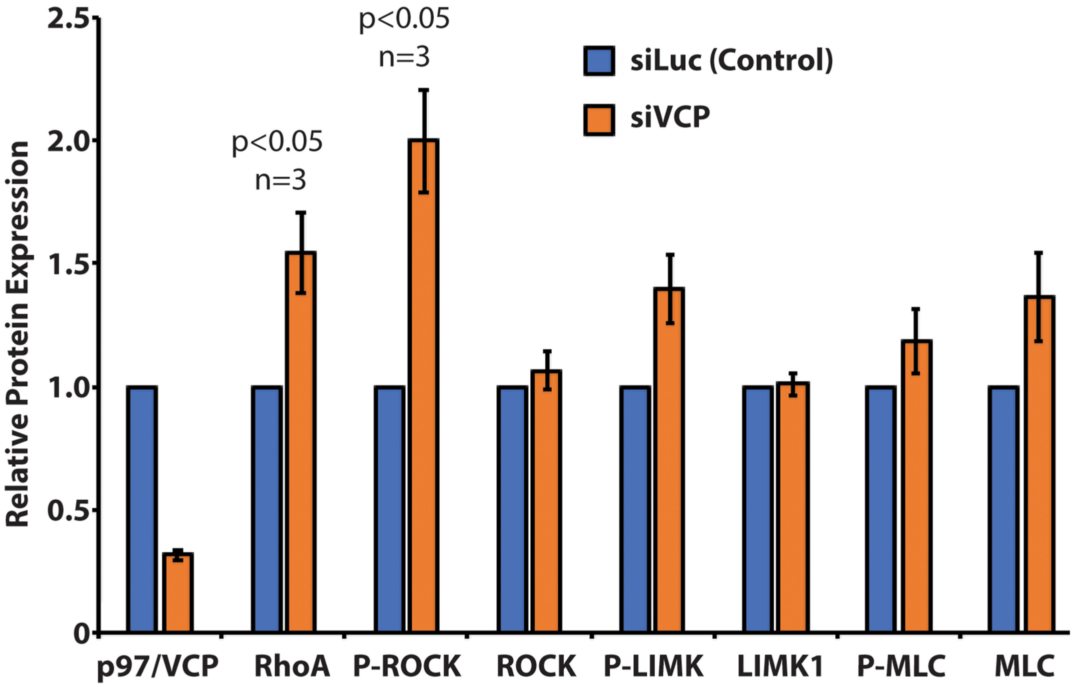

Supplementary Figure 4: The densitometric analysis of the immunoblots of the Rho-ROCK pathway proteins in control and p97/VCP knockdown U-2 OS cells. Upon knockdown of p97/VCP, there were increase in protein expression of RhoA and phosphorylated ROCK proteins, *p*-value is indicated in the histogram and error bars show ± SEM). The protein expression levels were normalized with respect to the amount of actin.

Supplementary Movie 1: Live cell-imaging of control and siVCP knockdown U-2 OS cells showing the difference in actin dynamics in the presence and absence of p97/VCP. See Supplementary Movie 1
